# Electrocatalysis in MOF Films for Flexible Electrochemical Sensing: A Comprehensive Review

**DOI:** 10.3390/bios14090420

**Published:** 2024-08-28

**Authors:** Suyuan Zhang, Min Wang, Xusheng Wang, Jun Song, Xue Yang

**Affiliations:** 1 Sinopec (Shanghai) Research Institute of Petrochemical Technology Co., Ltd., Shanghai 201210, China; zhangsuy.sshy@sinopec.com; 2 School of Life Sciences, Shanghai University, Shanghai 200444, China; 3 School of Materials Science and Engineering, Zhejiang Sci-Tech University, Hangzhou 310018, China; xswang@zstu.edu.cn

**Keywords:** electrocatalysis, MOFs, film, flexible device

## Abstract

Flexible electrochemical sensors can adhere to any bendable surface with conformal contact, enabling continuous data monitoring without compromising the surface’s dynamics. Among various materials that have been explored for flexible electronics, metal–organic frameworks (MOFs) exhibit dynamic responses to physical and chemical signals, offering new opportunities for flexible electrochemical sensing technologies. This review aims to explore the role of electrocatalysis in MOF films specifically designed for flexible electrochemical sensing applications, with a focus on their design, fabrication techniques, and applications. We systematically categorize the design and fabrication techniques used in preparing MOF films, including in situ growth, layer-by-layer assembly, and polymer-assisted strategies. The implications of MOF-based flexible electrochemical sensors are examined in the context of wearable devices, environmental monitoring, and healthcare diagnostics. Future research is anticipated to shift from traditional microcrystalline powder synthesis to MOF thin-film deposition, which is expected to not only enhance the performance of MOFs in flexible electronics but also improve sensing efficiency and reliability, paving the way for more robust and versatile sensor technologies.

## 1. Introduction

Flexible electronic devices, characterized by intrinsic flexibility and adaptability, adhere effortlessly to any bendable surface, ensuring highly consistent contact with attached objects [1,2,3,4]. This unique capability enables continuous and stable monitoring and transmission of sensor data without restricting dynamic activities. Such attributes are particularly crucial in wearable technology, where devices must closely conform to the human body, adjusting flexibly with movement and contours to maintain optimal functionality [5,6,7]. Among various sensors, electrochemical sensors stand out for their ability to convert chemical information into electrical signals, facilitating the rapid and precise detection of target analytes [8,9,10,11,12]. These sensors exhibit high flexibility and bendability, seamlessly adapting to complex and irregular surfaces while enduring various mechanical stresses [13,14]. Consequently, electrochemical sensors are indispensable in wearable devices [15,16,17], environmental monitoring [18,19,20], and healthcare diagnostics [21,22,23]. The sensitivity, stability, and selectivity of electrochemical sensors critically depend on the working electrode, a core component influencing sensor performance [24,25,26,27]. The rapid evolution of nanomaterials such as carbon nanotubes [28,29], semiconductor metal oxides [30,31], porous framework compounds [32,33], and conductive polymers [34,35] has significantly enhanced sensor sensitivity by facilitating electron transfer and signal response intensity. This advancement expands the applications of electrochemical sensors in biological detection, environmental monitoring, and medical diagnostics.

Metal–organic frameworks (MOFs) are crystalline materials formed by coordinating metal ions or clusters with organic ligands, renowned for their high surface area, tunable porosity, and multifunctional chemical properties [36,37,38,39]. These attributes make MOFs suitable for diverse applications, including gas storage [40,41], catalysis [42,43], and sensing [44,45]. MOFs exhibit dynamic responses to physical and chemical stimuli, making them particularly attractive for integration into large-area flexible electronic devices that adapt to environmental changes, offering more accurate and reliable sensing capabilities [46,47]. Although MOFs are generally electrically insulating, they can be electrocatalytic because of their high specific surface area, tailorable structure including hybrid, composite, and post-synthetic chemical modification, and thermal conversion. However, traditional methods such as pressing or drop-casting MOF powders onto working electrodes suffer from issues such as uneven coating, poor continuity, and susceptibility to detachment, compromising electrode activity distribution and consequently lowering sensor sensitivity and accuracy, especially in detecting low-concentration analytes [1,48,49,50]. Moreover, discontinuous electrode coatings may render portions of the electrode surface inactive, further diminishing sensor performance. Addressing these challenges involves developing uniform and dense MOF film electrodes to enhance sensor performance by maximizing active site exposure and optimizing electron transfer efficiency within the film [51,52,53].

**Figure 1:**
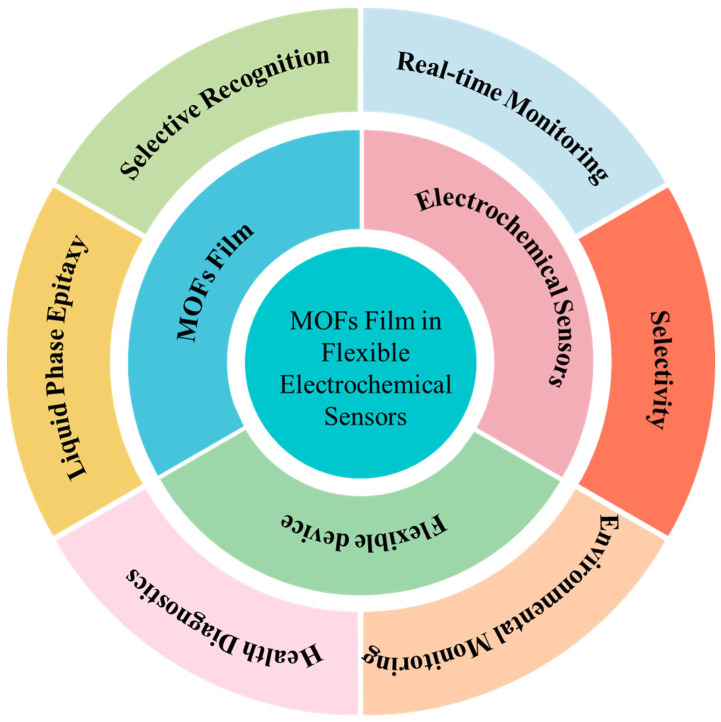
Overview of MOF films in flexible electrochemical sensors.

Integrating MOF thin films into flexible electrochemical sensors represents a frontier in sensor technology, promising enhanced sensitivity, selectivity, and durability. This review summarizes recent advancements and challenges in MOF thin films applied in flexible electrochemical sensors (Figure 1). By exploring the structure–performance relationships and assembly protocols of MOF-based sensing electronic devices, this review emphasizes their multifunctionality and effectiveness in healthcare, environmental monitoring, and food safety applications. In environmental monitoring, MOF-based flexible sensors demonstrate high sensitivity and specificity for detecting pollutants, toxins, and other harmful substances. In food safety, MOF-based sensors ensure food safety and quality by detecting contaminants and indicators of spoilage. Furthermore, this review summarizes the latest progress achieved by MOF-based flexible electronic technologies in monitoring vital signs, detecting biomarkers, and contributing real-time data for disease diagnosis and management in healthcare in a timely manner. Key challenges such as stability, reproducibility, and scalability are discussed alongside future directions for MOF-based flexible sensor development, ranging from advanced manufacturing techniques to personalized health monitoring and large-scale environmental sensing. This review aims to bridge the gap between MOF research and their integration into flexible electronic sensing, providing valuable insights for developing high-performance flexible sensing devices through MOF integration.

## 2. Flexible Electrochemical Sensors

Enhancing the mechanical properties of flexible electrochemical sensors is essential for ensuring their durability, reliability, and performance under diverse conditions. Creating multi-layered structures with alternating stiff and soft layers offers a balanced approach to achieving both mechanical strength and flexibility. Additionally, designing the sensor’s surface with micro or nanostructures can enhance mechanical robustness by distributing stress more evenly across the sensor. Applying protective coatings, such as parylene or other elastomeric materials, further improves the mechanical durability of the sensor surface, shielding it from wear and tear. Surface plasma treatment can also enhance adhesion between layers or between the sensor material and the substrate, thereby improving the overall mechanical integrity. By judiciously applying these methods, the mechanical properties of electrochemical sensors can be enhanced significantly, ensuring their long-term performance and reliability, particularly in wearable and flexible electronics.

### 2.1. Applications of Flexible Electrochemical Sensors

**Figure 2:**
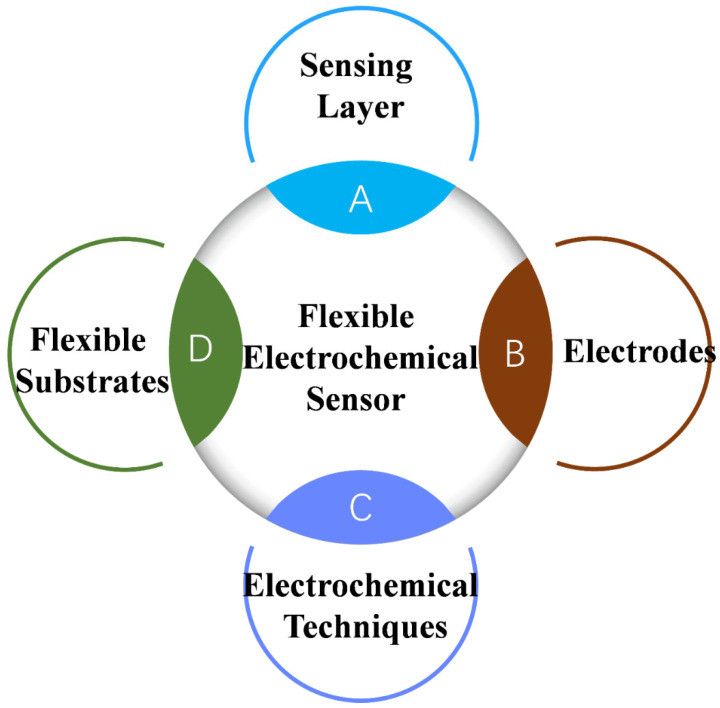
Schematics illustrating a flexible electrochemical sensor composed of substrates, electrodes, electrochemical techniques, and sensing layers.

As shown in Figure 2, flexible electrochemical sensors, a rapidly evolving field at the intersection of materials science, electronics, and analytical chemistry, are designed to bend, stretch, and conform to various shapes while maintaining functionality. These characteristics make them ideal for a wide range of applications, including wearable technology, medical diagnostics, environmental monitoring, and industrial uses. The development of flexible matrix materials has led to the emergence of flexible electrochemical sensors that align with these trends [54,55,56]. Dissimilar to traditional electrochemical sensors, which are typically rigid, incorporating flexible and stretchable materials extend their application scope. Flexible electrochemical sensors have the potential to revolutionize various fields with their unique combination of flexibility, sensitivity, and versatility [57,58,59]. To meet the demands of emerging signals and specialized environments, sensor technology must continue to develop. This includes the development of new materials, innovative processes, and novel types of sensors, as well as advancements in sensor integration and intelligence. Flexible electrochemical sensors should also possess qualities such as transparency, flexibility, ductility, and the ability to bend or fold freely, making them portable and wearable [60,61,62].

Flexible electrochemical sensors are indispensable in various aspects of life, such as monitoring physiological parameters to detect potential health issues at an early stage. Ongoing advancements in materials science, fabrication techniques, and electronic system integration will continue to enhance the capabilities and applications of flexible electrochemical sensors [63,64]. Flexible electrochemical sensors are poised to become essential tools for future technologies in health monitoring, environmental protection, and industrial process control. Despite operating on the same basic principles as traditional electrochemical sensors—utilizing the redox reactions of analytes at the sensor’s surface—the key difference lies in their ability to maintain performance while deformed. In summary, flexible electrochemical sensors are crucial in various fields because of their sensitivity, selectivity, and real-time monitoring capabilities [65,66,67]. Ongoing research focuses on enhancing sensor performance, expanding their range of applications, and addressing current challenges to meet the growing demand for reliable chemical detection technologies [68,69,70].

### 2.2. Materials Employed in Electrochemical Sensing Platforms

Electrochemical sensors operate based on the principle of electrochemistry, which involves the interaction between electrical energy and chemical reactions occurring at electrode surfaces. A typical flexible electrochemical sensing platform comprises several key components: the substrate, the active layer, and the interface layer. The selection of materials for these components is critical, as they must maintain their functional integrity under mechanical deformation, such as bending and stretching. For wearable applications, biocompatibility is essential to prevent adverse reactions when in contact with skin or other biological tissues [71,72,73]. Additionally, the materials used must exhibit chemical and thermal stability to ensure consistent sensor performance over time and under various environmental conditions. These materials also need to be compatible with microfabrication techniques, allowing for seamless integration into flexible electronics [74,75].

#### 2.2.1. Substrate Layer

The substrate layer is a foundational component of flexible electrochemical sensors, providing structural support and mechanical flexibility. The substrate must withstand bending, stretching, and other forms of mechanical deformation without compromising its structural integrity or sensor performance. For wearable applications, the substrate must be biocompatible to ensure it does not cause adverse reactions when in contact with skin or other biological tissues. Additionally, the substrate should be chemically inert and stable under a range of environmental conditions to maintain sensor performance over time. It must also be suitable for advanced microfabrication processes, enabling the seamless integration of various sensor components and materials. For example, Avuthu et al. [76] made a flexible electrochem sensor screen printed on polyethylene terephthalate that selectively detected the heavy metal ions Hg^2+^ and Pb^2+^. Salem et al. [77] reported a flexible poly(imide) film substrate applied to a laser-induced graphene-patterned electrode of a sensitive, selective, and reproducible electrochemical sensor for the detection of nitrite in water. The choice of substrate material depends on the specific application requirements, including the desired flexibility, biocompatibility, and environmental stability needed for the sensor’s intended use. Flexible substrates are often made from materials such as polyethylene terephthalate (PET), polyimide (PI), or polydimethylsiloxane (PDMS), as these materials provide the necessary flexibility and mechanical stability [78,79,80,81].

#### 2.2.2. Electrode Layer

The electrode layer is a critical component of flexible electrochemical sensors, serving as the site for electrochemical reactions and signal transduction [82,83]. It typically consists of conductive materials that maintain their performance under mechanical deformation, such as bending and stretching. The electrode material must be chemically stable and resistant to corrosion to ensure long-term performance in diverse environmental conditions while also possessing high electrical conductivity to facilitate efficient electron transfer during sensing. The active layer includes electrodes made from materials such as gold, platinum, carbon-based materials (graphene and carbon nanotubes), or conducting polymers (PEDOT and polypyrrole (PPy)) [26,84,85,86,87]. Electrochemical sensors typically incorporate working electrodes, reference electrodes, and sometimes auxiliary electrodes [88]. The working electrode facilitates the chemical reaction of interest, where interactions with the target analyte lead to changes in electrochemical properties, such as potential or current. These changes translate into measurable electrical signals, such as current, potential (voltage), or impedance. Materials such as conductive polymers (e.g., polyaniline, PPy, and PEDOT), carbon-based materials (e.g., graphene, CNTs, and carbon black), metal nanoparticles and nanowires (e.g., gold, silver, and platinum), and metal oxides [89,90,91] (e.g., ITO and ZnO) are chosen for their conductivity, flexibility, and compatibility with microfabrication techniques.

In summary, designing the electrode layer of flexible electrochemical sensors requires balancing conductivity, mechanical flexibility, biocompatibility, chemical stability, and integration capability to optimize performance in diverse applications, especially in wearable sensing devices.

#### 2.2.3. Interface Layer

The interface layer in flexible electrochemical sensors serves a critical role in ensuring optimal performance and durability [92,93]. It acts as a bridge between the active electrode material and the external environment, influencing both the sensor’s sensitivity and its mechanical properties. This layer promotes strong adhesion between the active electrode material and the substrate, ensuring the sensor remains intact during mechanical deformation such as bending or stretching, which is crucial for maintaining electrical continuity and preventing delamination. Efficient charge transfer between the active electrode material and the analyte in the environment is facilitated, thereby enhancing the sensor’s sensitivity and response time. The optimization of the interface layer often involves adjusting its surface morphology and chemical composition. Additionally, the interface layer serves as a barrier, protecting the active electrode material from environmental factors such as moisture, contaminants, and chemical reactions that could degrade sensor performance over time [94,95].

For manufacturing flexibility, the interface layer must be compatible with microfabrication techniques, ensuring reliable and efficient deposition or patterning on flexible substrates. It can incorporate hydrogel coatings, ionic liquids, or nanostructured materials to improve sensor–biofluid interactions, enhancing sensitivity and selectivity by facilitating efficient mass transport and providing a biocompatible environment [96,97,98,99]. In summary, the interface layer in flexible electrochemical sensors plays a crucial role in optimizing performance, ensuring durability under mechanical stress, enhancing sensitivity, providing protection from environmental factors, and promoting biocompatibility for wearable applications.

#### 2.2.4. Active Layer

The active layer in flexible electrochemical sensors plays a crucial role in facilitating electrochemical reactions for detecting and quantifying target analytes. This layer is composed of materials selected for their specific electrochemical properties, including conductivity, catalytic activity, and selectivity towards the analyte of interest. Popular materials include conductive polymers such as polyaniline, polypyrrole, and poly(3,4-ethylenedioxythiophene) (PEDOT), known for their excellent electrical conductivity and stability. Carbon-based materials such as graphene and carbon nanotubes offer high surface area, conductivity, and chemical stability, enhancing sensitivity and response times. Metals such as gold, platinum, and silver nanoparticles provide catalytic activity, while metal oxides such as tin dioxide (SnO_2_), titanium dioxide (TiO_2_), and indium oxide (In_2_O_3_) offer both conductivity and catalytic properties for diverse sensing applications [100,101,102]. Hybrid materials combining these elements can further tailor properties such as selectivity and stability to specific sensing needs. Deposition onto a flexible substrate through techniques such as spin-coating, inkjet printing, or vapor deposition ensures uniform coverage and adherence [103,104,105,106,107,108]. The composition and structure of this layer are critical in determining sensor sensitivity, selectivity, and overall performance, making material selection and deposition methods crucial areas of research and development in flexible electrochemical sensor technology.

## 3. MOFs in Flexible Electrochemical Sensors

### 3.1. MOFs as a Tunable Platform for Flexible Electrochemical Sensors

**Figure 3:**
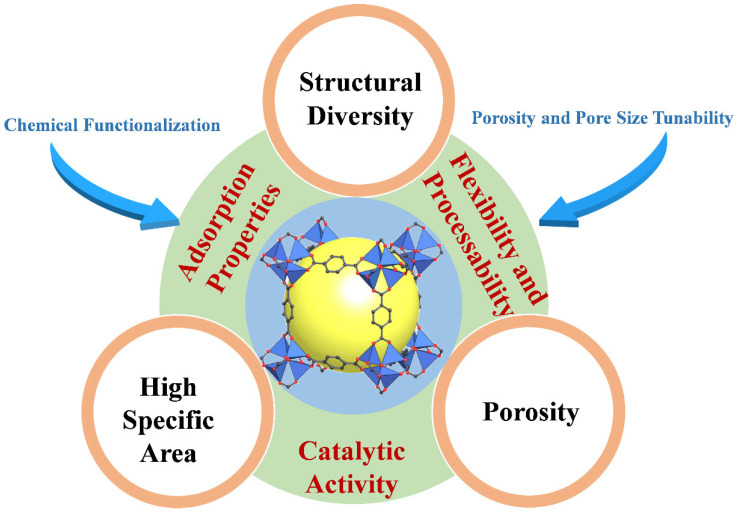
Fundamental MOF properties related to sensing applications.

MOFs are an extraordinary class of porous materials that have garnered significant attention in past decades because of their unique and highly tunable properties. Key findings highlight the unique properties of MOFs, such as high surface area, tunable pore structures, and chemical functionality, which contribute to enhanced sensitivity, selectivity, and stability in electrochemical sensing applications [109,110,111]. MOFs provide a large surface area per unit volume, maximizing the active sites available for electrochemical reactions, thereby enhancing sensor sensitivity. The porosity of MOFs can be finely tuned by selecting specific metal ions and organic ligands, allowing for precise control over analyte diffusion and interaction within the sensor. Furthermore, MOFs can be designed with different metal nodes and organic linkers, offering flexibility to optimize sensor selectivity and stability toward target analytes. They can also be functionalized with various functional groups, biomolecules, or nanoparticles to impart additional functionalities such as enhanced catalytic activity or specific analyte recognition [112,113,114]. In addition to their functional versatility, some MOFs exhibit excellent chemical stability, which is crucial for sensor longevity and reliability, especially in dynamic environments, including wearable sensors. Certain MOFs also possess inherent catalytic activity, enabling them to directly participate in electrochemical reactions or enhance the catalytic performance of other sensor materials. The ability of MOFs to integrate into flexible substrates, such as polymer matrices or films, further enables the fabrication of flexible and conformable sensors suitable for wearable applications. Researchers are actively exploring strategies to leverage these advantages of MOFs, such as hybridizing them with conductive polymers or incorporating them into nanocomposites, to significantly enhance sensor performance in terms of sensitivity, selectivity, and stability [115,116]. As shown in Figure 3, ongoing research focuses on optimizing MOF properties, deposition techniques, and integration strategies to enhance sensor performance, broaden applications, and address challenges such as mechanical durability and biocompatibility for wearable sensor technologies [83,117].

### 3.2. Selective Recognition of MOFs for Electrochemical Sensing

Selective recognition refers to the ability of a material to specifically adsorb, detect, or react with a class of molecules or ions in a complex mixture environment. In sensing technology, selective recognition is particularly important because there are a lot of interferences in the actual environment. This ability not only helps quickly and accurately identify the target molecule or ion but also avoids interference from other unrelated substances, ensuring the accuracy and reliability of data. MOFs are highly designable and functional materials, and their selective recognition ability is a basic aspect of MOF function [118,119]. This ability is mainly due to the high porosity, tunable structure, and chemical versatility of MOFs. These properties allow MOFs to interact strongly with specific molecules or ions, enabling efficient selective recognition.

The selective recognition ability of MOFs has shown great application potential in many fields. In the field of gas separation, MOFs can selectively adsorb and separate specific gas molecules, such as carbon dioxide and hydrogen [120,121]. In the field of sensing, the high surface area and adjustable pore size of MOFs enable them to effectively bind biomarkers such as uric acid, glucose, and ascorbic acid, enabling the rapid detection of these substances [122,123]. In the field of catalysis, the specific structure and chemical properties of MOFs enable them to selectively catalyze certain chemical reactions, improving reaction efficiency and product purity. In addition, MOFs have shown promising applications in environmental remediation, such as the selective adsorption and removal of harmful substances from water [124].

Taking the application of MOFs in the field of electrochemical sensors as an example, their wide surface area and porosity provide sufficient space for preferentially adsorbing neurotransmitters such as dopamine [125]. Due to the differences in structure and properties between dopamine and other neurotransmitters, MOFs can selectively interact with dopamine to achieve efficient detection. In addition, the aperture of MOFs can be precisely controlled during synthesis to achieve size-selective identification of guest molecules of different sizes and shapes [126].

It is worth mentioning that the selective recognition ability of MOFs can be fine-tuned by adjusting their pore size and surface chemistry [127,128]. This tunability makes MOFs more flexible and adaptable in sensor applications. By precisely controlling the structure and chemistry of MOFs, we can achieve efficient selective identification of different target molecules, leading to more efficient and targeted applications in a variety of scientific and industrial fields. With the continuous development and progress of science and technology, the research on the selective recognition ability of MOFs will continue to deepen. Future research will likely reveal more information about the selective recognition mechanism of MOFs and further optimize their structure and function for more efficient and accurate sensor applications. This will bring more convenience and possibilities to our lives and promote the development of science and technology to a higher level.

## 4. Fabrication of MOF Films

In flexible electrochemical sensors, MOF films serve as the active layer where electrochemical reactions take place, detecting analytes through changes in electrical signals. MOF films can be deposited onto flexible substrates using techniques such as the drop-casting method, allowing for the fabrication of flexible sensors that can conform to irregular surfaces or be integrated into flexible electronics [129,130]. Recently, there has been a growing emphasis on integrating high-quality MOF films with flexible substrates, which opens up new possibilities for flexible and wearable electronics, sensors, and other advanced applications. MOF thin films possess a substantial specific surface area, abundant metal active sites, and an orderly and compact arrangement, rendering them promising for applications in thin film separation, sensors, and devices. Currently, various methods are available for synthesizing MOF thin films, including in situ growth, interface assembly, thermal stamping, hydrothermal deposition, secondary growth, and electrochemical approaches. These techniques enable the preparation and functionalization of MOF thin films while establishing a solid foundation for their utilization in thin film devices. However, controlling the thickness, surface morphology, and growth orientation of MOF thin films remains challenging, restricting their application in high-quality, demanding devices such as separation processes [131]. Consequently, there is an urgent need to develop a method that can produce MOF thin films with uniform flat surfaces and specific growth orientations. The insolubility of MOFs in any solvent makes the preparation of thin films a great challenge.

Flexible substrates, such as polymers (e.g., polyimide, PET), elastomers (e.g., PDMS), and even paper, offer unique advantages in terms of mechanical flexibility, lightweight nature, and conformability to irregular surfaces. The fabrication of MOFs on flexible substrates represents a promising frontier in materials science, offering new opportunities for flexible electronics, wearable sensors, and advanced functional materials. Continued advancements in fabrication techniques, materials design, and application-specific integration will further propel the field toward practical implementations in various industrial and consumer applications. As research progresses, the synergy between MOFs and flexible substrates is expected to drive innovations in flexible electronics and beyond, paving the way for transformative technologies in the coming years.

### 4.1. Substrate Treatment

**Figure 4:**
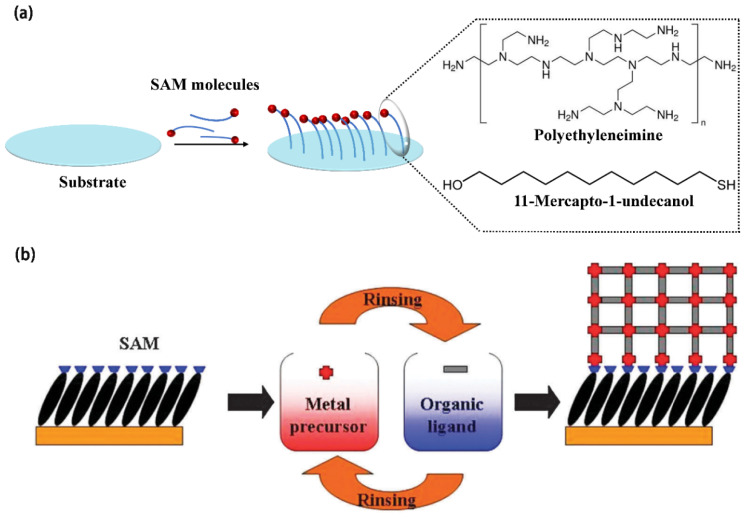
(**a**) Hydrophilic treatment of the substrate. (**b**) A schematic diagram illustrating the step-by-step approach for growing MOFs on substrates functionalized with self-assembled monolayers (SAMs) (O. Shekhah, Layer-by-Layer method for the synthesis and growth of surface-mounted metal–organic frameworks (SURMOFs). Reproduced with permission [132].

Ensuring a strong and reliable interface between MOF films and flexible substrates is crucial for practical applications, as a mismatch in mechanical properties can lead to delamination or performance degradation [132]. The surface of the substrate used for MOF film preparation must be thoroughly cleaned to remove contaminants such as dust, grease, and organic residues. Common cleaning methods include solvent cleaning (using solvents such as acetone, ethanol, or isopropyl alcohol) and ultrasonic cleaning to ensure a pristine surface. Additionally, to enhance adhesion between the MOF film and the substrate, the substrate surface needs to undergo hydrophilic treatment by introducing functional groups (Figure 4). Self-assembled monolayers (SAMs) with specific functional groups are particularly beneficial as they can modify the chemical properties of the substrate surface, influencing the growth of MOF crystals and the properties of the resulting film. For conductive glass, silicon wafers, sheet metals, and other planar substrates, this is typically achieved by treating them with a piranha solution (a mixture of concentrated sulfuric acid and 30% hydrogen peroxide in a volume ratio of 3:1) heated to 80–100 °C for at least half an hour. This treatment modifies the substrate surface with a layer of hydroxyl groups, facilitating the coordination of metal ions and thereby enhancing the adhesion between the MOF film and the substrate. Therefore, select the appropriate substrate material, and through the steps of cleaning, treatment, and surface modification, ensure the flatness of the substrate surface and appropriate surface chemical properties to promote the growth and adhesion of MOF films.

### 4.2. Liquid Phase Epitaxy

Liquid phase epitaxy (LPE) is a method used to sequentially assemble materials layer-by-layer on the surface of a substrate [133]. High-quality MOF films produced by LPE on the substrate surface enable precise control over their crystal structure and thickness. Dip-coating, spin-coating, and layer-by-layer deposition are common techniques where flexible substrates are immersed or coated with MOF precursor solutions. Liquid phase epitaxy offers several advantages for preparing MOF films: it operates under relatively mild conditions, facilitates uniform growth of high-quality films over large substrate areas, and allows precise control over the crystal structure, pore size, and chemical functionality of MOFs. This capability is beneficial for maximizing the exposure of MOF active sites, thereby meeting specific application requirements for MOF materials.

**Figure 5:**
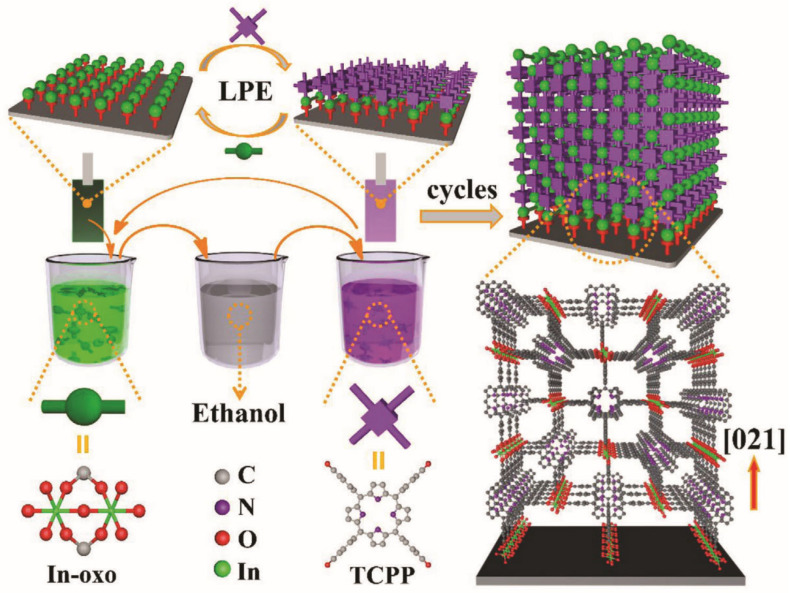
Schematic illustration of porphyrinic MOF thin film grown on a functionalized substrate using liquid phase epitaxy [133].

As shown in Figure 5, LPE primarily includes the following steps: first, metal ions and organic ligands are fully dissolved in their respective solvents to form precursor solutions. The concentration of these solutions is typically adjusted to control the thickness of the desired MOF film during growth. Following substrate pretreatment, the precursor solution is applied to the substrate surface. By varying the concentration, temperature, and duration of exposure to metal ions and organic ligands in the solution, MOF crystals grow layer-by-layer from the substrate surface. After each immersion, the substrate is thoroughly cleaned with ethanol and dried with nitrogen to remove any residual reactants. Upon completion of MOF film growth, additional steps such as solvent removal, enhancement of crystallinity, or surface modification are performed to refine the structure and properties of the MOF films further.

### 4.3. In Situ Growth Methods

In situ growth methods involve direct synthesis of MOF films on the relevant substrates, often using solvothermal or microwave-assisted methods to induce MOF formation directly on the substrate surface. The hydrothermal/solvothermal method is a significant approach for preparing MOF films [134,135]. This method involves exposing the substrate directly to a precursor sol or solution under either hydrothermal or solvothermal conditions, facilitating the nucleation and growth of MOF crystals directly on the substrate. However, this approach presents several challenges and limitations. To address issues such as uneven deposition or overly thick film layers, two strategies are commonly employed: orienting the substrate with the crystal growth face down or vertically immersing it into the reaction solution. Both methods help enhance the uniformity and control the thickness of MOF films to some extent. Nevertheless, despite these measures, MOF films obtained directly via hydrothermal/solvothermal methods often exhibit poor orientation and limited controllability over their properties. This instability can make it challenging to regulate the film properties precisely according to specific requirements. Effective surface functionalization of the substrate material is crucial to improving the quality of MOF films.

**Figure 6:**
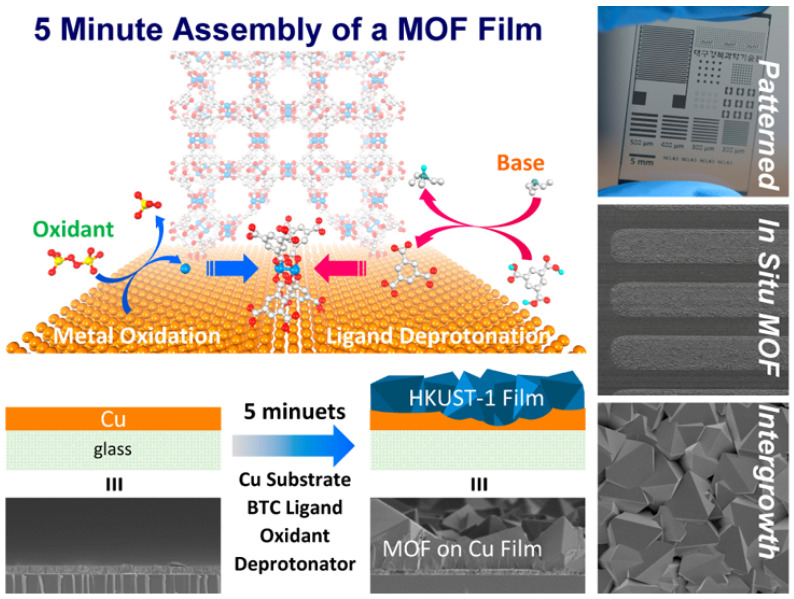
Rapid growth of HKUST-1 Films on Cu substrates. Reproduced with permission [138].

In addition to surface functionalization, another method involves depositing synthesized powdered MOFs directly onto the substrate using surface drop-coating or spin-coating with MOF particle suspensions or mixtures with solutions such as Nafion [136,137]. This approach allows for rapid solvent evaporation to achieve precise control over the thickness and uniformity of films. It also enables adjustment of MOF particle concentration and type to tailor the film performance as needed. In conclusion, while the hydrothermal/solvothermal method is effective for MOF film preparation, careful attention is required to enhance film uniformity, thickness control, orientation, and overall controllability of film properties. Surface functionalization and the use of MOF particle suspensions offer promising avenues to enhance the quality and performance of MOF films further. In a study by Jeong Liu et al. [138], HKUST-1 films were rapidly grown on Cu substrates, where the Cu substrate acts both as a conducting substrate and a source of Cu^2+^ ions during the synthesis of films (Figure 6).

MOF films not only retain the inherent characteristics of MOFs, such as a large specific surface area, abundant metal active sites, and high porosity, but also exhibit significant potential applications in gas separation, sensors, heterogeneous catalysis, and other fields. However, the majority of MOFs exist in powder form and are insoluble in any solvent, posing challenges to their transformation into films. Despite successive research advancements in methods, including in situ growth, interface assembly, hot pressing, hydrothermal deposition, secondary growth, and electrochemical deposition for preparing MOF thin films, controlling the thickness, surface morphology, and growth orientation remains difficult. This limitation hinders their application in high-quality devices and separation technologies. Hence, there is an urgent need to develop methods that can produce MOF films with uniform surfaces and specific orientations.

### 4.4. Electrophoretic Deposition Method

**Figure 7:**
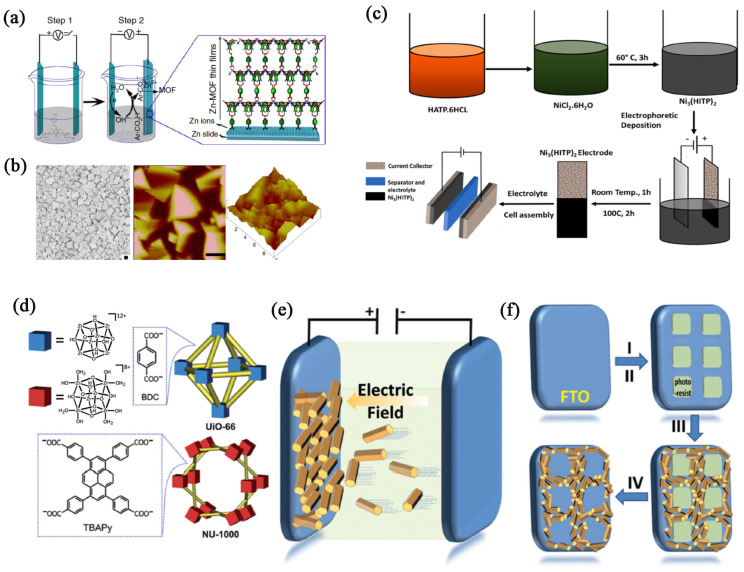
(**a**) Diagram illustrating the electrochemical formation of MOF films. (**b**) SEM image alongside a representative height AFM image. Scale bar, 1 mm. Reproduced with permission [139]. (**c**) Diagram illustrating the fabrication process and electrophoretic deposition of Ni_3_(HITP)_2_ [140]. (**d**) Components and illustrations of the crystal structures of UiO-66 and NU-1000 include Zr-containing nodes. (**e**) A diagram demonstrating the principle of MOF film growth via electrophoretic deposition. (**f**) Illustration of the MOF EPD film patterning procedure. (I) spin coating photoresist on FTO, (II) using photolithography to create patterned squares of photoresist, (III) deposition of MOF particles, and (IV) removal of the remaining photoresist squares to obtain the desired pattern. Reproduced with permission [141].

Electrophoretic deposition (EPD) is a versatile method for fabricating MOF films on various substrates. It involves the migration of charged MOFs in a suspension under the influence of an electric field, leading to their deposition on an electrode. EPD is a promising technique for creating MOF films, particularly for applications requiring high surface area, controlled porosity, and specific chemical functionalities. For example, Cao et al. [139] introduce a straightforward method for depositing interpenetrated, crystalline MOF films onto conductive metal-plate anodes through an in situ electrochemical assembly process. The thickness and uniformity of the MOF film are well controlled by the temperature, reaction time, and voltage (Figure 7a,b).

EPD can produce uniform and dense films, which are essential for various applications. This technique is particularly advantageous for creating uniform and well-adhered MOF films, which are useful in applications such as sensors, catalysis, and separation membranes. As illustrated in Figure 7c, the creation of an ultra-high cycling stability supercapacitor involves the use of electrophoretic deposition (EPD), employing the 2D MOF Ni_3_(HITP)_2_ as the active material in the electrodes [140]. The electrophoretic deposition produced highly dense films, resulting in a Ni_3_(HITP)_2_ supercapacitor achieving an exceptionally high areal specific capacitance of 15.69 mF cm^−2^. This is the highest capacitance value recorded for MOFs to date.

The first step involves preparing a stable colloidal suspension of the desired MOF particles (Figure 7d,e) [141]. The MOF particles can be synthesized beforehand and dispersed in a suitable solvent. Stabilizers or surfactants may be added to prevent aggregation of the MOF particles and to ensure a stable suspension. Two electrodes are used, typically made of conductive materials such as metal or conductive glass (Figure 7f). The substrate on which the MOF film is to be deposited serves as the working electrode, while the counter electrode completes the circuit. An electric field is applied between the electrodes. The charged MOF particles migrate towards the electrode with the opposite charge, where they accumulate and form a film. However, precise control over film thickness can be challenging and requires careful optimization of deposition parameters. Maintaining a stable suspension of MOF particles is crucial for consistent film quality. The charge of the MOF particles affects their mobility in the electric field and must be controlled for effective deposition.

### 4.5. Polymer-Assisted Strategy for Creating MOF Films

**Figure 8:**
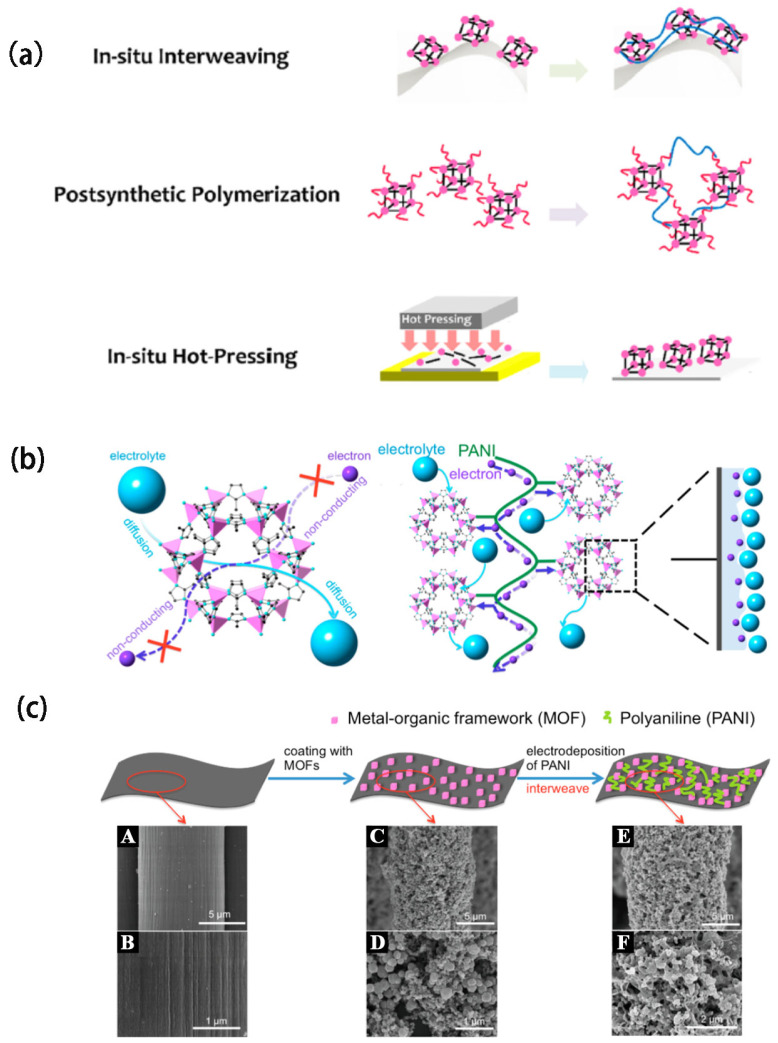
(**a**) Three strategies for the fabrication of MOF films. Reproduced with permission [145]. (**b**) Schematic Representation of ZIF-67 onto carbon cloth and further electrically deposited PANI to give a flexible conductive porous electrode [65]. (**c**) Schematic illustration of the two-step fabrication process and SEM images of MOFs interwoven by electrochemically-deposited PANI f (**A**,**B**) the carbon clothfibers, (**C**,**D**) after coating with ZIF-67, and (**E**,**F**) after electropolymerization of anilin. Reproduced with permission [146].

Polymers such as polyvinyl alcohol (PVA), poly(methyl methacrylate) (PMMA), or polydopamine (PDA) serve as a stabilizer or binder and help in controlling MOF film growth [142,143,144]. The polymer is dissolved in a solvent that can effectively dissolve it without affecting the MOF precursors. The polymer concentration is adjusted to achieve the desired viscosity and film properties. The polymer solution is applied onto the substrate using techniques such as spin-coating, dip-coating, or spray-coating. The MOF precursor solution is introduced onto the polymer-coated substrate. This can be accomplished by sequential application or co-dissolution, depending on the method used. This polymer-assisted strategy can help achieve uniform and high-quality MOF films with tailored properties, making it useful for various applications in materials science and engineering. Wang et al. [145] proposed three strategies for MOF film fabrication (Figure 8a). For example, a novel approach has been developed to reduce the bulk electrical resistance of MOFs by incorporating polyaniline (PANI) chains into the MOF structure. As shown in Figure 8b, this is achieved through the electrochemical deposition of PANI onto the MOF crystals [65]. In this method, cobalt-based MOF crystals, specifically Zeolitic Imidazolate Framework-67 (ZIF-67), were first synthesized onto a carbon cloth (CC) substrate [65]. Subsequently, PANI was electrochemically deposited onto the ZIF-67, resulting in the creation of a flexible, conductive porous electrode, designated as PANI-ZIF-67-CC (Figure 8c). This strategy effectively maintains the integrity of the underlying MOF structure while enhancing the electrical conductivity. Electrochemical evaluations reveal that the PANI-ZIF-67-CC electrode exhibits an exceptional areal capacitance of 2146 mF cm^−2^ at a scan rate of 10 mV s^−1^, demonstrating its high performance and potential for advanced applications.

## 5. Application of MOF-Based Flexible Electrochemical Sensors

MOF-based electrochemical sensors have promising applications across several domains because of their unique properties and capabilities. MOF-based electrochemical sensors represent a versatile and promising technology with significant potential to address critical challenges in environmental monitoring, healthcare diagnostics, food safety, and industrial processes. Continued advancements in MOF materials and sensor design are essential to realize their full impact across these diverse application areas.

### 5.1. Environmental Monitoring

**Figure 9:**
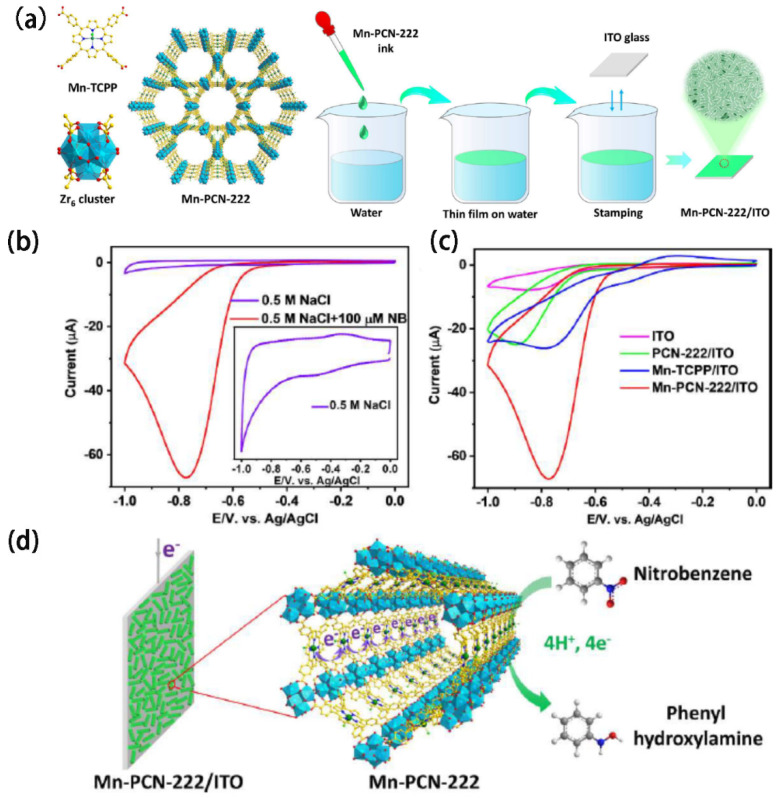
(**a**) Diagram illustrating the film of Mn-PCN-222 deposited on a conductive indium tin oxide (ITO) surface. (**b**) Cyclic voltammetry (CV) profiles of the Mn-PCN-222/ITO electrode in a 0.5 M NaCl solution, both with and without 100 μM NB. (**c**) CV curves obtained from different electrodes focusing on the reduction of 100 μM NB. (**d**) Schematic representation of the possible mechanism for NB reduction on the Mn-PCN-222/ITO electrode. Reproduced with permission [148].

MOFs can be used to remove contaminants from water, including heavy metals, organic pollutants, and radionuclides [147]. Their large surface area and functional groups enable them to adsorb and trap a wide range of pollutants. In a study by the Roland A. Fischer group [148], the porphyritic MOF Mn-PCN-222 was deposited onto a conductive ITO surface (Figure 9). The high surface area of Mn-PCN-222/ITO as a working electrode supports elevated current densities, facilitating highly sensitive analyses toward nitrobenzene (NB). The metalloporphyrin center in Mn-PCN-222 enables analyte-specific redox catalysis, allowing for the simultaneous detection of multiple analytes in binary and ternary systems. This capability makes this MOF film effective for detecting a wide range of trace pollutants with a high sensitivity under real-world conditions. MOF films can be easily integrated with flexible electronic substrates, such as conductive polymers or graphene-based materials, allowing for the development of flexible, wearable sensors for detecting pollutants and hazardous substances in air and water. In summary, MOF films combine flexibility with high sensitivity and tunable properties, making them a promising material for advanced electrochemical sensors in environmental monitoring applications.

### 5.2. Health Diagnostics

**Figure 10:**
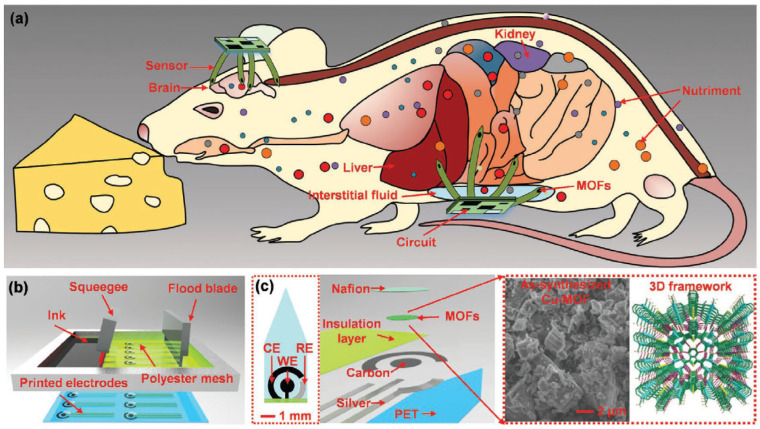
Schematics of MOF-based flexible electrochemical sensors for implant detection. (**a**) The flexible MOF-based sensors. (**b**) The screen-printing process of flexible sensors. (**c**) Multilayer structure of the MOFs electrochemical sensor. Reproduced with permission [158].

In this regard, flexible sensors provide real-time monitoring of various physiological parameters such as heart rate [149,150], blood pressure [151,152], and biomarkers [153,154], offering accurate and reliable data support for healthcare and fitness applications. These data aid precise health assessments by medical professionals and empower fitness enthusiasts to understand their physical condition for scientifically informed exercise routines better. Electrochemical biosensors monitor biomolecular reactions at electrode surfaces, enabling real-time detection of biomarkers, drugs, and disease indicators crucial for early diagnosis and health management [155,156,157]. However, integrating MOFs with flexible electronic devices for wearable sensing poses challenges because most MOFs have inherently low electrical conductivity, which limits their effectiveness in electronic applications. Conductive MOFs represent a burgeoning class of multifunctional materials characterized by abundant catalytic active sites, highly porous structures, and intrinsic conductivity. These attributes make them highly desirable for electrochemical sensing applications. As shown in Figure 10, Ling et al. [158] demonstrated multichannel implantable electrochemical sensors that were surface-modified with conductive copper-MOFs and cobalt-MOFs. Experiments with live cells and animals indicate that these MOF-modified sensors are biologically safe for cells and capable of detecting l-Trp in both blood and interstitial fluid. This research represents the first effort to integrate MOF films with flexible sensors to achieve highly specific and sensitive implantable electrochemical detection.

**Figure 11:**
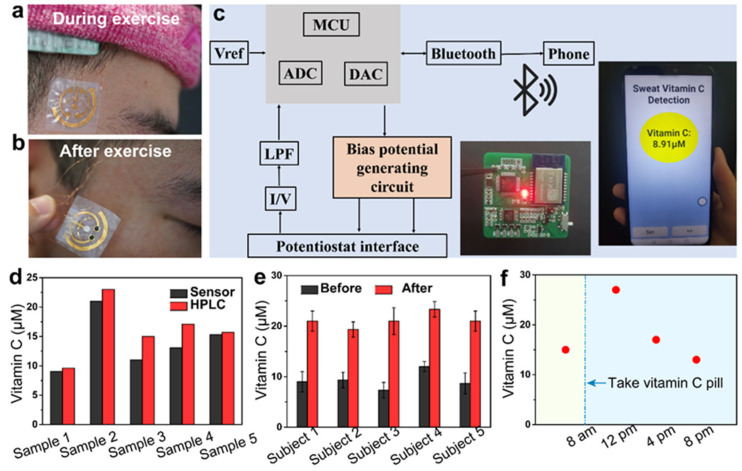
Demonstration of conductive MOF-based wearable sweat sensor for wireless ascorbic acid analysis. (**a**,**b**) Photograph of the sensor during and after aerobic exercise, respectively. (**c**) Illustration of the wireless transmission components. (**d**) Comparison of the vitamin C concentrations of sweat samples detected using the sweat sensor and HPLC. (**e**,**f**) Comparison of the sweat vitamin C levels of five subjects before and after vitamin C intake. Reproduced with permission [159].

Conductive MOFs offer a lot of potential for advancing wearable sensors, especially with ongoing research and development aimed at overcoming these challenges. As shown in Figure 11, Yang et al. [159] demonstrated the development of bacterial nanocellulose (BNC)-based wearable sensors that utilize conductive MOFs as the sole electrode material for sweat sensing. The MOF-based layered film electrodes, characterized by inherent conductivity, permanent porosity, and tunable catalytic properties, enable selective and simultaneous monitoring of nutritional and metabolic biomarkers. Additionally, BNC serves as a highly skin-adherent sensor platform, providing excellent permeability to the underlying skin, which facilitates natural sweating and evaporative cooling—key factors for wearable comfort and the prolonged use of epidermal electronics. The successful demonstration of a wireless system capable of continuously monitoring dynamic trends in sweat vitamin C following supplement intake highlights the potential monitoring biomarkers in bodily fluids for early disease detection.

### 5.3. Food Safety

**Figure 12:**
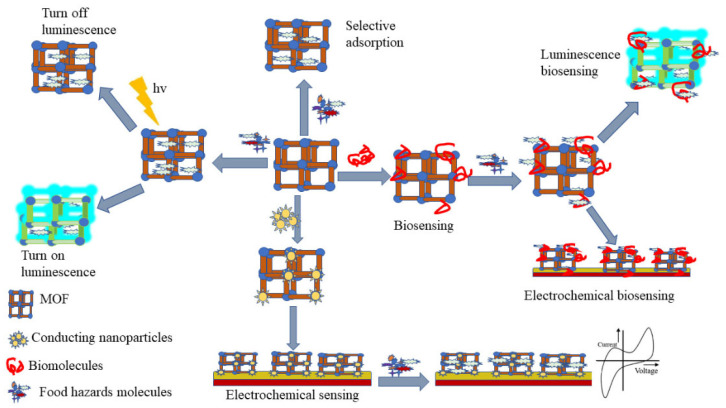
Schematic representation of MOF-based sensors for food safety. Reproduced with permission [160].

In the context of food safety, MOF-based flexible electrochemical sensors have gained significant attention because of their potential to detect harmful substances and contaminants efficiently [160]. These sensors can play a crucial role in ensuring the safety and quality of food products by identifying contaminants such as heavy metal ions, pesticide residues, adulterants, and indicators of spoilage. MOFs can be engineered to selectively bind heavy metal ions such as lead, cadmium, and mercury, enabling the detection of trace amounts of these harmful metals in food products. Additionally, certain MOFs can interact with organophosphates and other pesticide residues, facilitating their detection in agricultural produce. For detecting adulterants, such as melamine in milk or Sudan dyes in spices, MOF-based sensors can identify specific chemical signatures associated with these substances (Figure 12). Moreover, the freshness of perishable goods can be monitored by detecting volatile organic compounds (VOCs) released during spoilage. MOFs can adsorb these VOCs, leading to measurable changes in the sensor’s electrical properties [161,162]. However, for these sensors to be widely adopted in the food industry, they must meet regulatory standards for food safety testing. While they show promise in laboratory settings, scaling up the production of MOF-based sensors for commercial applications poses challenges. Ensuring the long-term stability and durability of MOFs in different environments is also crucial, as these factors can impact sensor performance over time.

In summary, MOF-based flexible electrochemical sensors hold significant promise for enhancing food safety by providing rapid and accurate detection of contaminants and pathogens. Ongoing advances in materials science and engineering are likely to improve their performance and expand their range of applications, further supporting food safety efforts.

## 6. Challenges and Future Directions

### 6.1. Challenges

Preparing flexible electrochemical sensors based on MOF films is a challenging field involving multiple aspects. First, a core challenge lies in the preparation and control of high-quality MOF films. Despite various methods, such as atomic layer deposition, electrochemical methods, and cross-linking-induced assembly strategies being available, each method has its limitations, particularly in precisely controlling the morphology, orientation, and thickness of MOF films. Second, flexible electrochemical sensors need to have good electrical conductivity. Generally, MOFs exhibit poor conductivity, significantly restricting their utilization in electrochemical sensors. While conductivity can indeed be enhanced through compositing with conductive materials such as metal particles and carbon nanotubes, such a process may compromise the surface area of MOFs, ultimately impacting their sensing capabilities. Third, the stability of MOF films presents another significant issue, encompassing chemical stability in electrochemical environments and mechanical stability against bending and stretching. Ensuring the effective integration of MOF films with flexible substrates such as polymers or carbon-based materials requires consideration of compatibility, adhesion, and optimization of process parameters.

Additionally, practical challenges in real-world applications include sensor reproducibility, long-term stability, and cost-effectiveness. MOFs are sensitive to environmental factors such as humidity, temperature, and pH, which can impact the stability and performance of sensors. MOFs could work in mildly acidic environments (pH 4–7) and neutral to slightly basic conditions (pH 7–9). Thus, the phosphate-buffered saline (PBS, pH = 7.4) and Tris-HCl (pH 7–9) buffers are ideal for MOF-based electrochemical sensing. Maintaining the structural integrity of MOF films under mechanical stress (bending and stretching) is crucial for flexible sensors. Ensuring durability without compromising performance poses a significant challenge. Achieving consistent quality and reproducibility in MOF film synthesis is difficult but crucial for reliable sensor performance.

Moreover, the instability of MOFs in aqueous environments is double-edged for their electrochemical sensing application as a flexible device. Some researchers have tried to avoid or improve this scenario via methods such as protective encapsulation, chemical modification, and producing composites with water-stable materials. Other researchers applied the partial degradation of MOFs in water to expose more active sites or create defect sites that enhance sensor sensitivity. This controlled degradation can be beneficial for electrochemical sensing applications where high sensitivity is required. The high chemical sensitivity of MOFs in water can also be regarded as a fast response time for sensing.

Researchers are actively exploring new preparation methods and processes to address these challenges, optimize sensor structure and performance, and seek practical solutions for applications. These efforts include developing protective coatings to enhance stability, utilizing advanced manufacturing technologies for precise MOF film production, and developing wireless communication systems for real-time data transmission and remote monitoring. Addressing these challenges and exploring future directions is critical for advancing the field of flexible electrochemical sensors integrated with MOF films. Continued research and development in these areas will lead to more robust, reliable, and versatile sensors capable of meeting diverse application needs, from healthcare diagnostics to environmental monitoring. Developing cost-effective, scalable methods for producing high-quality MOF films suitable for commercial applications remains challenging. Many MOFs have poor conductivity, limiting their application in electrochemical sensors. Improving the conductivity of MOF films without sacrificing their intrinsic properties is a challenge. Effectively integrating MOF films with flexible substrates and electronic components into signal processing and data transmission remains a technical barrier. Achieving high selectivity for specific analytes while minimizing interference from complex matrices is challenging. For many applications, such as medical diagnostics and environmental monitoring, improving the sensitivity of MOF-based sensors to detect analytes at extremely low concentrations is necessary. Ensuring that MOF films maintain their performance over long periods, especially under harsh conditions, is crucial for practical applications.

### 6.2. Future Directions

Electrochemical sensors, renowned for their exceptional accuracy and reliability, are essential tools in environmental and healthcare applications. MOFs, with their high porosity, low density, and large surface area, offer outstanding performance in areas such as gas adsorption and ion exchange. Integrating MOF thin films with flexible electrochemical sensors opens new possibilities for enhanced detection accuracy and sensitivity, presenting broad prospects for future development. With rapid advancements in nanotechnology and continuous improvements in measurement techniques, flexible electrochemical sensors integrated with MOF thin films show immense potential for applications in sectors such as biomedicine, smart homes, and intelligent transportation. To enhance the stability of MOF materials without compromising their performance, developing protective coatings or surface treatments is crucial. Additionally, combining MOF thin films with other flexible substrate materials, such as polymers or carbon-based materials, not only improves mechanical strength and flexibility but also offers greater customization possibilities for sensors.

Advanced manufacturing technologies such as 3D printing and micromachining enable the production of complex and precise MOF thin films on flexible substrates. The optimization of in situ growth techniques allows for better control over film morphology, orientation, and thickness, thereby enhancing reproducibility and scalability. Additionally, surface functionalization of MOF films by introducing specific functional groups improves selectivity toward target analytes significantly. The integration of conductive materials such as graphene or conductive polymers further enhances the electrical and overall performance of sensors. Looking ahead, the integration of MOF-based sensors with flexible and stretchable electronic devices is set to usher in a new era of seamless incorporation into wearable technologies. Concurrently, the development of wireless communication systems will facilitate real-time data transmission and remote monitoring, revolutionizing fields such as environmental monitoring and traffic management. Designing MOF thin films capable of detecting multiple analytes simultaneously will enhance sensor versatility. Furthermore, research into self-healing materials will help extend the lifespan of flexible sensors, contributing to more durable and reliable devices.

In personalized health monitoring, customized MOF-based sensors can detect specific biomarkers related to individual health conditions, facilitating early disease diagnosis and treatment. Furthermore, networks of MOF-based sensors can be deployed for large-scale environmental monitoring, providing real-time data on pollution levels and climate conditions. Currently, electrochemical sensors are extensively used in environmental protection, food safety, pharmaceuticals, and the chemical industry. The incorporation of MOF thin films will further expand their application scope, especially in scenarios that demand high sensitivity and selectivity. As artificial intelligence and automation technologies continue to advance, flexible electrochemical sensors integrated with MOF thin films are expected to become smarter and more automated. They will be capable of automatically identifying and quantifying multiple chemical substances, as well as providing real-time monitoring and data transmission. With the escalating interest in health, environmental protection, and related fields, MOF-based flexible electrochemical sensors exhibit vast potential for applications in these domains. Specifically, these sensors offer significant value in areas such as environmental monitoring, medical diagnosis, and food safety, among others. Continuous technological innovation and upgrades will enable MOF-based flexible electrochemical sensors to achieve groundbreaking advancements in manufacturing processes, signal transmission methodologies, and sensitivity enhancements. Consequently, this will establish a stronger foundation for the large-scale production and widespread implementation of sensors. In conclusion, these sensors will play critical roles across various fields, significantly contributing to human well-being and economic development. We anticipate ongoing progress and innovation in this technology, heralding new advancements in precision detection and monitoring.

**Figure 13:**
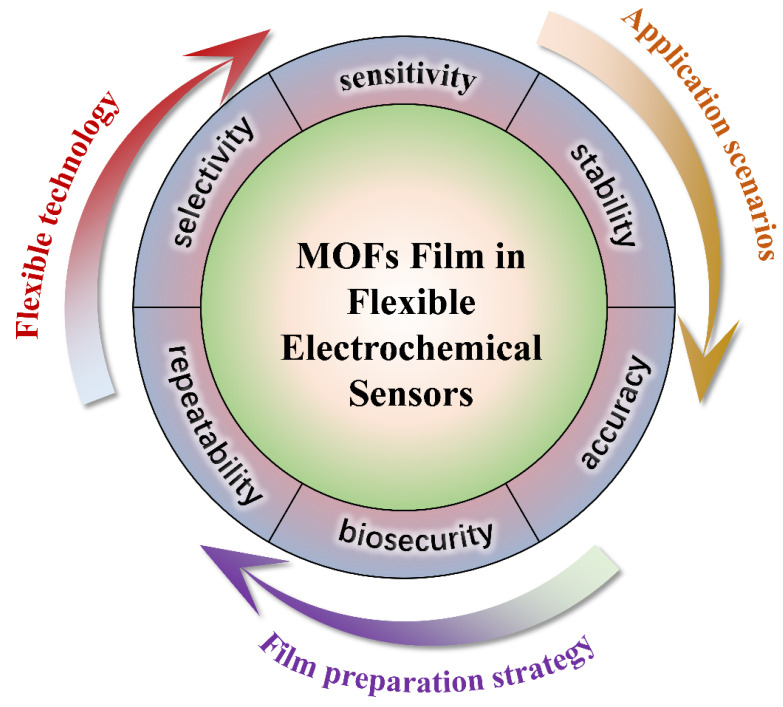
An overview of MOF films for flexible electrochemical sensing.

As shown in Figure 13, the future of MOF-based flexible electrochemical sensing is promising, with the potential to revolutionize various fields by providing more precise, portable, and adaptable sensing solutions. MOF-based sensors can be developed for rapid, on-site diagnostics, particularly in resource-limited settings. These portable, flexible devices could provide quick and reliable results for various medical conditions. Future devices could integrate MOF-based sensors directly onto the skin, providing continuous monitoring of health parameters without the need for invasive procedures. Embedding MOF-based sensors into textiles could lead to clothing that responds to environmental changes or the wearer’s physiological state, providing data to wearable electronics or directly alerting the wearer. Flexible MOF-based sensors could be integrated into energy storage devices, such as batteries or supercapacitors, to monitor their performance and health, enabling more efficient and safer energy systems.

## 7. Conclusions

MOFs offer significant potential in the field of flexible electrochemical sensors because of their unique properties, such as high surface area, adjustable porosity, and chemical versatility. These attributes can greatly enhance sensor performance in terms of sensitivity, selectivity, and durability, making MOFs ideal for a wide range of applications, including environmental monitoring and biomedical diagnostics. In this review, we summarize the research progress on flexible electrochemical sensors based on MOFs and discuss the challenges and key technical issues in this area. Although considerable efforts have been made to produce high-quality MOF films and integrate them with flexible electronics technology for use in flexible sensors, several challenges remain. While various methods, such as atomic layer deposition and electrochemical techniques, can be employed to fabricate MOF films, these approaches have their own advantages and limitations, making it difficult to precisely control film morphology, orientation, and thickness simultaneously. Flexible electronic devices must endure mechanical deformations such as bending and stretching during use, necessitating MOF films with excellent mechanical stability. Ensuring compatibility between MOF films and common flexible substrates, such as polymers and carbon-based materials, is crucial to prevent delamination or detachment during operation. Additionally, when MOFs serve as the active layer in sensors, they must provide both high sensitivity and selectivity to detect target substances accurately. For practical applications, MOF-based flexible electronic devices need to demonstrate good repeatability and long-term stability to ensure accurate and reliable measurements. Dissimilar to well-established materials such as silicon and conventional semiconductors, the fundamental charge transport and photonic properties of MOFs are not yet fully understood. Future research must address current challenges, such as improving the stability, repeatability, and scalability of MOF thin film deposition. Moreover, exploring new methods for integrating MOFs with advanced sensor technologies could expand the boundaries of sensor design and functionality. By unlocking the full potential of MOFs in sensor applications, researchers can develop innovative solutions that meet emerging societal and technological needs.
